# A new species of *Diochus* from Baltic amber (Coleoptera, Staphylinidae, Diochini)

**DOI:** 10.3897/zookeys.138.1896

**Published:** 2011-10-19

**Authors:** Stylianos Chatzimanolis, Michael S. Engel

**Affiliations:** 1 Department of Biological and Environmental Sciences, University of Tennessee at Chattanooga, 615 McCallie Ave, Dept. 2653, Chattanooga, Tennessee 37403, USA; 2 Division of Entomology (Paleoentomology), Natural History Museum, and Department of Ecology & Evolutionary Biology, 1501 Crestline Drive – Suite 140, University of Kansas, Lawrence, Kansas 66045, USA

**Keywords:** Tertiary, Eocene, Lutetian, fossil, Staphylininae, Diochini, taxonomy

## Abstract

The first fossil of the staphylinine tribe Diochini Casey is described and figured from an inclusion in mid-Eocene (Lutetian) Baltic amber. *Diochus electrus* **sp. n.** is distinguished from its congeners and the diversity of rove beetles (Staphylinidae s.l.) is summarized briefly.

## Introduction

More so than any other amber deposit in the world, the fossiliferous resin from the blaue Erde of northern Europe has garnered the attention of researchers, artists, and amateurs. For literally millennia Baltic amber has been the focus, if not obsession, of innumerable individuals and as such its included flora and fauna is one of the most completely understood paleoecosystems. Despite this fascination and intense activity, there remains huge swaths of the fauna to revise and newly document. Among those groups requiring significant attention are the beetles of the family Staphylinidae (sensu Bouchard et al. 2011). Most species, largely of the subfamilies Scydmaeninae and Pselaphinae, were described more than a century ago by (Schaufuss (1888, 1890a, 1890b, 1890c, 1892, 1896) and are in need of revision and figuring, should new material eventually be located (*vide* Appendix). Fortunately, several new works during the last 35 years, particularly the last decade, have added significantly to this fauna and provided a more modern perspective on staphylinid diversity in Baltic amber (*vide* Appendix). Unfortunately, the diverse subfamily Staphylininae has not been recorded formally since Schaufuss (1888) described *Bembicidiodes inaequicollis*, a species more recently considered of uncertain subfamilial affinity (Herman 2001).

In this paper we describe the first fossil species of *Diochus* Erichson from middle Eocene Baltic amber and as the first, definitive fossil staphylinine. The tribe Diochini Casey includes the genera *Antarctothius* Coiffait and Saiz, *Coomania* Cameron, and *Diochus*. The tribe has not received much taxonomic attention and the boundaries between these genera are not clear. Newton (1985) suggested that *Antartoctothius* might be co-generic with *Diochus*, which is the genus with the highest number of species (40) in the tribe. *Diochus* has a worldwide distribution but the majority of species are found in the New and Old World tropics. There are ten species of *Diochus* in the Palearctic region (Smetana 2004; western Palearctic species revised by Assing 2003) and only one in the Nearctic (Smetana 1982). Smetana (1982) noted that *Diochus* is in dire need of systematic revision and that it is extremely hard to differentiate between species.

## Material and methods

Measurements were made using an ocular micrometer on an Olympus SZX-12 stereomicroscope and all measurements refer to maximum width or length of a particular structure. Total length is measured from the anterior margin of the clypeus to the posterior margin of abdominal segment VIII. Due to the placement of the fossil in amber, not all typical measurements were possible. Photomicrographs where prepared with a Nikon D1x digital camera attached to an Infinity K-2 long-distance microscope lens.

The age, origin, and biotic diversity of Baltic amber has recently been summarized by Weitschat and Wichard (2010). Material discussed herein is deposited in the Fossil Insect Collection of the Division of Entomology, University of Kansas Natural History Museum, Lawrence, Kansas, USA.

## Systematic placement

The fossil is placed in the tribe Diochini (and the genus *Diochus*) based on the following characters (from Smetana 1982): antennae not geniculate; maxillary palpus (P_2_ and P_3_) finely pubescent; neck narrow, only about a forth as wide as head and frons between antennal insertions truncate. The direct comparison of the fossil described here with *Coomania* was not possible due to the lack of *Coomania* specimens, however, in the published description of *Coomania* (Cameron 1939) the neck is much narrower than in *Diochus*, only a fifth as broad as the head.

## Systematic paleontology

**Family Staphylinidae Latreille, 1802**

**Subfamily Staphylininae Latreille, 1802**

**Tribe Diochini Casey, 1906**

**Genus *Diochus* Erichson, 1839**

### Diochus electrus

Chatzimanolis & Engel sp. n.

urn:lsid:zoobank.org:act:C24A1C8A-B27B-48EC-8100-2FE43C4913E6

http://species-id.net/wiki/Diochus_electrus

Figs 1–3 

#### Holotype.

**Figures 1–3.**
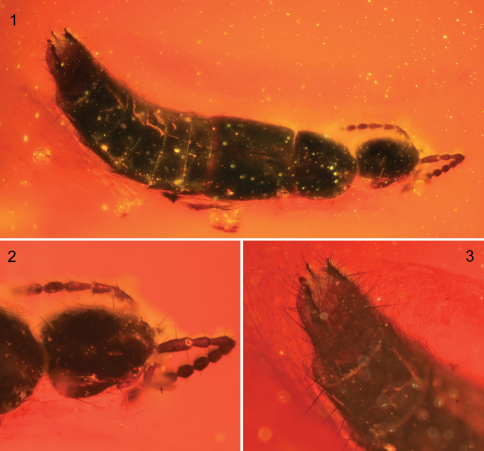
Photomicrographs of holotype female of *Diochus electrus* Chatzimanolis & Engel, sp. n. (B-244). **1** Dorsal view **2** Details of head **3** Details of abdominal apex.

 ♀; KU-NHM-ENT, B-244 (Fig. 1); with labels: “Amber: Baltic, middle Eocene (Lutetian), blaue Erde, Northern Europe, KU-NHM-ENT-B244” // “HOLOTYPE *Diochus electrus* Chatzimanolis and Engel, des. Chatzimanolis and Engel 2011”. Deposited in Fossil Insect Collection, Division of Entomology, University of Kansas Natural History Museum, Lawrence.

#### Diagnosis. 

*Diochus electrus* can be distinguished from otherwestern Palearctic species of the genus by the differences in the relative proportion of elytra to pronotum (elytra longer than pronotum in *Diochus electrus*; shorter than elytra in other species) and the proportions of the head (head much more elongate in the extant species than in *Diochus electrus*).

#### Description. 

Total length 3.5 mm; body coloration brown to black except antennae somewhat orange and abdominal segment VIII light brown. Head ovoid, length 0.56 mm, width 0.48 mm, slightly longer than wide (Fig. 2); compound eye length 0.18 mm, postoccular region convex, about twice as long as compound eyes; head with large macrosetae near posterior margin; head with transverse microsculpture and sparse small punctures. Antennomeres 1–5 longer than wide; antennomeres 6–10 subquadrate, antennomere 11 longer than wide; antennomere 1 as long as twice length of antennomere 2; antennomere 3 1.5 times longer than antennomere 2; antennomere 4 slightly shorter than antennomere 3; antennomere 5 slightly shorter than antennomere 4; antennomeres 6–9 subequal in length; antennomere 10 slightly longer than previous antennomeres but shorter than antennomere 11. Mouthparts not visible except right maxillary palp; maxillary palpomere I (P_1_) not visible, P_2_ longer than wide, club-like, about as long as P_3_; P_3_becoming wider distally; P_4_ extremely small, slender, conical, about seven times smaller than P_3_. Pronotum subquadrate, wider than head; pronotal length 0.64 mm, width 0.49 mm; anterolateral corners curved ventrally and not visible from above; pronotum smooth with sparsely scattered small, shallow punctures.Elytra longer than pronotum; elytra length 0.75 mm, elytra width 0.67 mm; elytra with dense macrosetae, expanding posteriorly; elytra sculptured as on pronotum. Legs (forelegs not visible) with slender tibiaecovered in long spurs distally; tarsi elongate, metatarsi almost as long as metatibia; metatarsomeres I and II greatly expanded. Abdomen with dense macrosetae (Figs 1, 3); segment VI longer than preceding segments; segment VII about twice as long as segment V; sternum VIII without any secondary sexual structures.

#### Etymology.

 The specific epithet is an adjective derived from the Latin noun for amber (electrum).

## Supplementary Material

XML Treatment for Diochus electrus

## Appendix

Checklist of described Baltic (including Bitterfeld and Rovno) amber Staphylinidaearranged by subfamily (sensu Bouchard et al. 2011); older records also exist for the subfamilies Euaesthetinae, Micropeplinae, Osoriinae, Oxytelinae, Phloecharinae, Proteininae, and Scaphidiinae but based on unidentified or undescribed material (e.g., Klebs 1910; Larsson 1978; Spahr 1981, Puthz 2006, 2008).

Subfamily Aleocharinae Fleming

*Adinopsis groehni* Zerche, 1999

*Aleochara (Aleochara) baltica* Paśnik & Kubisz, 2002

*Atheta (Datomicra) jantarica* Paśnik, 2005

*Baltioligota electrica* Paśnik, 2005

*Dictyon antiquus* Paśnik & Kubisz, 2002

*Electrogymnusa baltica* Wolf-Schwenniger, 2004

*Leptusa (Protoleptusa) defuncta* Semenov et al. 2001 [Rovno]

*Phymatura electrica* Paśnik & Kubisz, 2002

Subfamily Omaliinae MacLeay

*Pseudolesteua insinuans* Schaufuss, 1890b

Subfamily Oxyporinae Fleming

*Oxyporus blumenbachii* Gravenhorst, 1806

Subfamily Paederinae Fleming

*Lathrobium ambricum* Paśnik & Kubisz, 2002

*Lathrobium balticum* Paśnik & Kubisz, 2002

*Lathrobium jantaricum* Paśnik & Kubisz, 2002

*Lathrobium succini* Paśnik & Kubisz, 2002

*Lathrobium (Palaeolobrathium) whitei* Abdullah & Abdullah, 1968

Subfamily Pselaphinae Latreille

*Barybryaxis lata* Schaufuss, 1890a

*Batrisus antiquus* Schaufuss, 1890a

*Batrisus pristinus* Schaufuss, 1890a

*Bythinus foveopunctatus* Schaufuss, 1890a

*Bythinus schaufussi* Reitter, 1891 (nom. n. *proB*. *caviceps* Schaufuss, 1890a)

*Bythinus tenuipes* Schaufuss, 1890a

*Bythinus typicus* Schaufuss, 1890a

*Ctenistodes claviger* Schaufuss, 1890a

*Cymbalizon tyroides* Schaufuss, 1890a

*Dantiscanus costalis* Schaufuss, 1890a

*Deuterotyrus redivivus* Schaufuss, 1890a

*Euplectus lentiferus* Schaufuss, 1890a

*Euplectus mozarti* Schaufuss, 1890a

*Euplectus quadrifoveatus* Schaufuss, 1890a

*Euspinoides glabrellus* Motschulsky, 1856

*Faronus porrectus* Schaufuss, 1890a

*Faronus tritomicrus* Schaufuss, 1890a

*Greys conciliator* Schaufuss, 1890a

*Hagnometopias pater* Schaufuss, 1890a

*Hetereuplectus retrorsus* Schaufuss, 1890a

*Monyx spiculatus* Schaufuss, 1890a

*Nugaculus calcitrans* Schaufuss, 1890a

*Nugator stricticollis* Schaufuss, 1890a

*Pammiges spectrum* Schaufuss, 1890a

*Pantobatrisus cursor* Schaufuss, 1890a

*Rybaxis glabrella* (Schaufuss, 1890a)

*Rybaxis patris* (Schaufuss, 1892)

*Rybaxis veterum* (Schaufuss, 1890a)

*Tmesiphoroides cariniger* Motschulsky, 1856

*Tychus avus* Schaufuss, 1890a

*Tychus radians* Schaufuss, 1890a

*Tyrus electricus* Schaufuss, 1890a

Subfamily Scydmaeninae Leach

*Aenictosoma doenitzi* Schaufuss, 1892

*Clidicus balticus* Schaufuss, 1896

*Cryptodiodon corticaroides* Schaufuss, 1890c

*Electroscydmaenus pterostichoides* Schaufuss, 1890c

*Euconnus fossilis* Franz, 1976

*Euconnus liedtkei* Franz, 1976

*Euconnus sucini* Franz, 1976

*Euconnus wunderlichi* Franz, 1983

*Hetereuthia elegans* Schaufuss, 1890c

*Heuretus coriaceus* Schaufuss, 1890c

*Neuraphes fossilis* Franz, 1983

*Palaeomastigus helmi* Schaufuss, 1890c

*Palaeothia tenuitarsis* Schaufuss, 1890c

*Scydmaenoides nigrascens* Motschulsky, 1856

*Semnodioceras halticaeforme* Schaufuss, 1890c

*Stenichnus (Cyrtoscydmus) capucinus* (Schaufuss, 1890c)

*Stenichnus (Cyrtoscydmus) carinulatus* (Schaufuss, 1890c)

*Stenichnus (Cyrtoscydmus) laticlavus* (Schaufuss, 1890c)

*Stenichnus (Cyrtoscydmus) titubans* (Schaufuss, 1890c)

Subfamily Staphylininae Latreille

*Bembicidiodes inaequicollis* Schaufuss, 1888 (subfamily questioned: Herman 2001)

*Diochus electrus* Chatzimanolis & Engel, sp. n.

Subfamily Steninae MacLeay

*Stenus (Hemistenus) priscus* Benick, 1943

*Stenus abraham* Puthz, 2010

*Stenus archetypus* Puthz, 2010

*Stenus atavus* Puthz, 2010

*Stenus avus* Puthz, 2010

*Stenus groehni* Puthz, 2010

*Stenus ketura* Puthz, 2010

*Stenus methusalem* Puthz, 2010

*Stenus noach* Puthz, 2010

Subfamily Tachyporinae MacLeay

*Bolitobius groehni* Schülke, 2000

*Palaeosepedophilus succinicus* Paśnik & Kubisz, 2002

*Sepedophilus balticus* Paśnik & Kubisz, 2002

*Tachyporus bicoloratus* Paśnik, 2005
